# The Number of Surgical Interventions and Specialists Involved in the Management of Patients with Neurofibromatosis Type I: A 25-Year Analysis

**DOI:** 10.3390/jpm12040558

**Published:** 2022-04-01

**Authors:** Chih-Kai Hsu, Rafael Denadai, Chun-Shin Chang, Chuan-Fong Yao, Ying-An Chen, Pang-Yun Chou, Lun-Jou Lo, Yu-Ray Chen

**Affiliations:** Department of Plastic and Reconstructive Surgery and Craniofacial Research Center, Chang Gung Memorial Hospital, Chang Gung University, Taoyuan 333, Taiwan; kkhsu0315@gmail.com (C.-K.H.); denadai.rafael@hotmail.com (R.D.); frankchang@cgmh.org.tw (C.-S.C.); chuanfongyao@gmail.com (C.-F.Y.); whysomimi@gmail.com (Y.-A.C.); lunjoulo@cgmh.org.tw (L.-J.L.); uraychen@cgmh.org.tw (Y.-R.C.)

**Keywords:** neurofibromatosis, craniofacial, surgical treatment, multidisciplinary team, plexiform neurofibroma

## Abstract

Objective: In this study, we aim to present a single institution’s 25-year experience of employing a comprehensive multidisciplinary team-based surgical approach for treating patients with NF-1. Summary Background Data: All patients (*n* = 106) with a confirmed diagnosis of NF-1 who were treated using a multidisciplinary surgical treatment algorithm at Chang Gung Memorial Hospital between 1994 and 2019 were retrospectively enrolled. Patients were categorized into groups according to the anatomy involved (craniofacial and noncraniofacial groups) and the type of clinical presentation (plexiform and cutaneous neurofibromas groups) for comparative analysis. Methods: The number of surgical interventions and number of specialists involved in surgical care were assessed. Results: Most of the patients exhibited craniofacial involvement (69.8%) and a plexiform type of NF-1 (58.5%), as confirmed through histology. A total of 332 surgical interventions (3.1 ± 3.1 procedures per patient) were performed. The number of specialists involved in surgical care of the included patients was 11 (1.6 ± 0.8 specialists per patient). Most of the patients (62.3%) underwent two or more surgical interventions, and 40.6% of the patients received treatment from two or more specialists. No significant differences were observed between the craniofacial and noncraniofacial groups in terms of the average number of surgical interventions (3.3 ± 3.2 vs. 2.7 ± 2.7, respectively) and number of specialists involved (1.7 ± 0.9 vs. 1.4 ± 0.6). Patients with plexiform craniofacial involvement underwent a significantly higher average number of surgical interventions (4.3 ± 3.6 vs. 1.6 ± 1.1; *p* < 0.001) and received treatment by more specialists (1.9 ± 0.9 vs. 1.2 ± 0.5; *p* < 0.001) compared with those having cutaneous craniofacial involvement. Conclusions: In light of the potential benefits of employing the multidisciplinary team-based surgical approach demonstrated in this study, such an approach should be adopted to provide comprehensive individualized care to patients with NF-1.

## 1. Background

Neurofibromatosis type 1 (NF-1), also known as von Recklinghausen disease, is a hereditary condition with a worldwide incidence of 1 per 2500 to 3000 that predisposes an affected individual to tumor development and affects the central and peripheral nervous systems [1]. It is caused by pathogenic variants in the *NF1* gene and is characterized by *café au lait* macules, skin-fold freckling, Lisch nodules, optic glioma, and distinctive osseous lesions (such as sphenoid dysplasia or thinning of the long bone cortex) [1,2,3,4,5,6]. Patients with NF-1 are prone to numerous peripheral nerve sheath tumors [3,4,5,6]. Cutaneous and plexiform neurofibromas can grow to a large size, which considerably affects quality of life and has psychosocial implications because of itchiness, function impairment, physical disfigurement, and pain [7,8].

The wide range of clinical manifestations in neurofibromas, with varying anatomical location, number, size, progression, recurrence, local invasion, and compression of vital structures, necessitates multidisciplinary treatment and follow-up [8]. Although clinical trials have investigated the efficacy of various drugs (e.g., sirolimus, imatinib, tipifarnib, and pirfenidone) to treat particular features of NF1, surgical resection remains the standard procedure for the management of cutaneous and plexiform neurofibromas [9,10,11,12]. However, no single surgical algorithm is available to help clinicians in addressing the complexity and full spectrum of NF-1 abnormalities.

Because of the complexity due to the many regions and systems that may be affected, we applied an evolving multidisciplinary model of care that involves a range of health care professionals working in coordination at our center to provide comprehensive surgical treatment to complex and challenging conditions in patients presenting with NF-1. This long-term single-center study reports 25 years of evolving experience in implementing a multidisciplinary team-based surgical treatment approach for NF-1 management.

## 2. Methods

### 2.1. Patient Selection

This observational retrospective study included all patients with NF-1 who were surgically treated at a single institution (Linkou/Taoyuan Chang Gung Memorial Hospital) between 1994 and 2019. Patients with a confirmed diagnosis of NF-1, according to the cardinal criteria of consensus from the National Institutes of Health, who underwent surgical treatment performed by our multidisciplinary team were included. The exclusion criteria were patients with incomplete registration of treatment course, patients who received surgical treatment at another institution during the follow-up period, and replicated cases in the database. A total of 169 patients matches our inclusion criteria, and 53 patients were excluded. The remaining 106 cases, aged from 2 to 74, were enrolled for subsequent analysis.

### 2.2. Data Collection and Stratification

Demographic (age and sex), clinical (types of clinical presentation, disease involvement, and malignant transformation), and surgical (numbers and types of specialties and procedures) data were verified through review of electronic medical records and clinical photographs. On the basis of the type of clinical presentation, patients were categorized into either the plexiform neurofibroma or the cutaneous neurofibroma group. According to the anatomical region involved in the disease, patients were categorized into either the craniofacial (skull, face, orbit, brain, and cranial nerve) or the noncraniofacial (neck, chest wall, mediastinum, trunk, and extremities) group.

This study was approved by the Institutional Review Board of Chang Gung Medical Foundation (approval 202000258B0) and conducted in compliance with the 1975 Declaration of Helsinki, as amended in 1983. Singed consent forms for further academic use and publications were obtained from patients prior to every clinical photograph, including all of the cases presented in this article.

## 3. Statistical Analysis

Descriptive analysis was performed, and the data are presented as mean ± standard deviation for metric variables and percentages for categorical variables. Data distribution was verified using the Kolmogorov–Smirnov test. Independent *t* tests were employed for comparative analysis (craniofacial versus noncraniofacial groups and plexiform versus cutaneous groups). Two-sided *p* values of <0.05 were considered statistically significant. All analyses were performed using IBM SPSS software v22.0 (IBM Corp., Armonk, NY, USA).

## 4. Results

A total of 106 patients (57 men; mean age at initial evaluation of 24.44 ± 14.18 years; mean follow-up period of 9.71 ± 6.24 years) with NF-1 were enrolled in this study. Most of the patients had craniofacial involvement (*n* = 74, 69.8%; Table 1) and histologically confirmed plexiform NF-1 (*n* = 62, 58.5%). Most of the patients (*n* = 47, 63.5%) with craniofacial involvement had a plexiform type of presentation.

A total of 332 surgical interventions (3.13 ± 3.05 (range, 1 to 19) procedures per patient) were performed. The number of specialties involved in surgical care of the included patients was 11 (1.57 ± 0.79 specialties (range, 1 to 4) per patient; Table 2). Most of the patients (62.3%) underwent two or more surgical interventions, and 40.6% of the patients received treatment by two or more specialists.

No significant differences were observed between the craniofacial and noncraniofacial groups in terms of the average number of surgical interventions (3.31 ± 3.21 vs. 2.72 ± 2.67, respectively; *p* = 0.362) or the number of specialists involved in surgical care (1.65 ± 0.85 vs. 1.38 ± 0.61; *p* = 0.065). Patients with plexiform craniofacial involvement had undergone a significantly higher average number of surgical interventions (4.28 ± 3.62 vs. 1.63 ± 1.08; *p* < 0.001) and had been treated by more specialists (1.91 ± 0.91 vs. 1.19 ± 0.48; *p* < 0.001) than those having cutaneous craniofacial involvement.

In our analysis, plastic surgeons and ophthalmologists were found to be the most common combination of specialists, being adopted to treat 10 patients. Other specialist teams comprised plastic surgeons, ophthalmologists, and neurosurgeons (employed to treat four patients); plastic surgeons and radiologists (employed to treat four patients); plastic surgeons, ophthalmologists, neurosurgeons, and radiologists (employed to treat three patients); plastic surgeons and neurosurgeons (employed to treat three patients); and plastic surgeons, ophthalmologists, and radiologists (employed to treat three patients).

## 5. Multidisciplinary Approach

**Figure 1:**
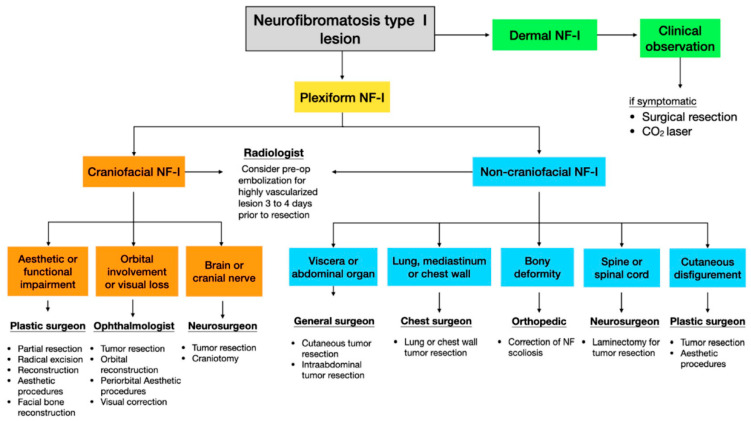
Multidisciplinary team-based treatment algorithm for neurofibromatosis type I (NF-1).

We have been using a comprehensive multidisciplinary team-based surgical approach in our center for managing patients with NF-1 for 25 years, with the approach evolving over time. Because of the progressive nature of NF-1 and the risk of it affecting multiple anatomical regions, the first professional who evaluates the patient has been responsible for general screening and referral to other specialists. Further referrals are made as needed according to further clinical findings made during the disease course. Additional professionals have been continually introduced when new clinical presentations are encountered by our team, and we have been updating the protocol on account of repeated observations of the same clinical presentation. At present, 11 specialties are engaged in providing a therapeutic algorithm at our center (Figure 1).

The psychological burden of a chronic and destructive disease associated with visible and stigmatizing skin lesions, which cause functional and aesthetic deficits in patients, is the main criteria for surgery. For cutaneous neurofibromas, which are characterized by superficial or dermal lesions, regular clinical follow-ups for the observation of each lesion is often sufficient [13]; surgical excision (scalpel- or laser-based removal) is indicated for symptomatic lesions (i.e., those exhibiting pain, bleeding, functional impairment, or disfigurement) or upon patient request [1,14,15]. For plexiform neurofibromas, which are characterized by deep lesions, the size, location, and symptomatic presentation serve as the influential factors to define the line of treatment. Targeted therapy can reduce tumor volume [16]; however, surgical excision remains the main therapeutic intervention for plexiform neurofibromas [8,15]. Imaging analysis (computed tomography, computed tomography angiography, and magnetic resonance imaging) can be used to help define the total size and depth of lesions as well as to reveal the involvement of adjacent structures; according to the imaging results, the appropriate specialist team can be assembled for surgical intervention [17].

## 6. Preoperative Embolization

**Figure 2:**
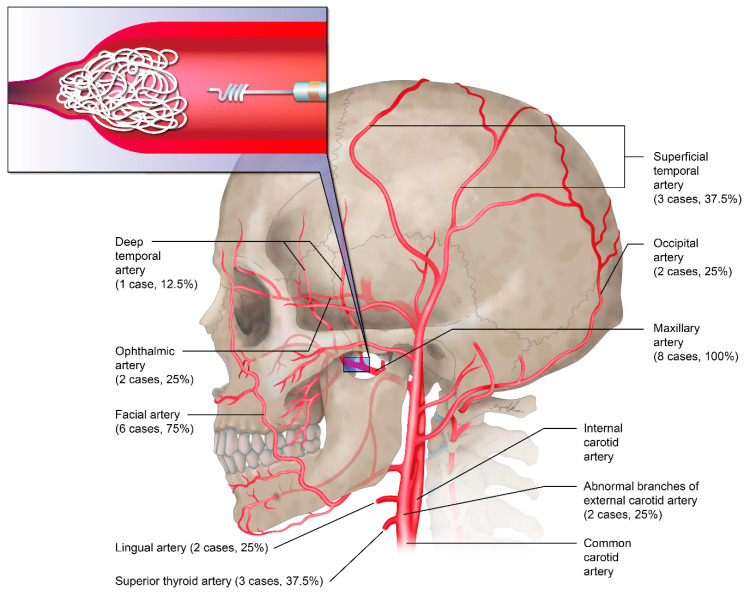
Target vessels of preoperative transarterial embolization (*n* = 8).

Before surgical excision of plexiform neurofibromas, the necessity for preoperative embolization should be considered. A total of three patients with craniofacial NF-1 and histologically confirmed plexiform neurofibromas received preoperative embolization by a radiology team before radical resection of neurofibromas to prevent massive intraoperative blood loss. Most of the embolization procedures were performed 3 or 4 days prior to surgery. All of the embolized vessels were branches of the external and internal carotid arteries, which included the maxillary artery (100%), facial artery (75%), superficial temporal artery (37.5%), and superior thyroid artery (37.5%; Figure 2).

The possible role of embolization should be evaluated whenever radical excision with massive bleeding is anticipated and should be re-evaluated before secondary surgical interventions in each patient. Despite successful preoperative embolization, massive blood loss occurred during surgical intervention in our experience, which highlights the need for pre-emptive anesthesia (regional blocks plus hypotensive anesthesia) and proper surgical execution with careful soft tissue manipulation, systematic ligation of vessels, and blood transfusions as needed.

## 7. Malignant Transformation

In total, 19 (17.9%) patients were diagnosed as having neurofibromatosis-related malignancy, which included malignant peripheral nerve sheath tumors (*n* = 17), gastrointestinal stromal tumor (GIST; *n* = 2), and cerebellar astrocytoma (*n* = 1); one patient exhibited both malignant peripheral nerve sheath tumor and GIST. Malignant peripheral nerve sheath tumor was evident in various anatomical regions, namely the spine (*n* = 6), lung (*n* = 4), trunk (*n* = 4), liver (*n* = 2), peritoneum (*n* = 2), extremities (*n* = 2), scalp/face (*n* = 2), kidney (*n* = 1), brain (*n* = 1), and jejunum (*n* = 1). Despite the postoperative adjuvant radiotherapy performed on the patients to reduce the risk of local recurrence and as a salvage strategy after tumor resection, a high recurrence rate and disease progression were observed.

## 8. Complicated Cases

**Figure 3:**
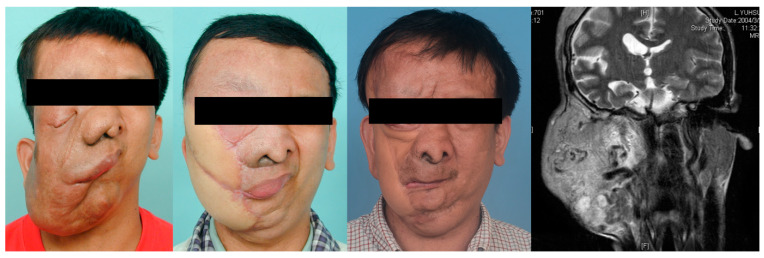
Clinical images of a 30-year-old man with hemifacial plexiform neurofibromatosis exhibiting no ipsilateral facial nerve function and vision. The patient was followed up by the multidisciplinary team for 12 years. Magnetic resonance imaging shows the extent of the plexiform tumor before surgical excision. Two sections of embolization of branches of the right external carotid artery, internal maxillary artery, and superficial temporal artery were completed before radical surgical excision. The distal right external carotid artery was ligated, and the tumor was extensively excised through scalp, preauricular, and submandibular incisions. Microsurgical free flaps (anterior lateral thigh flap and myocutaneous gracilis free flap) were transferred to reconstruct the facial defect. Additional procedures included orbitotomy, tumor resection (nasal and upper lip regions), labial suspension, canthopexies, eyelid reconstruction, and ocular prosthesis placement.

**Figure 4:**
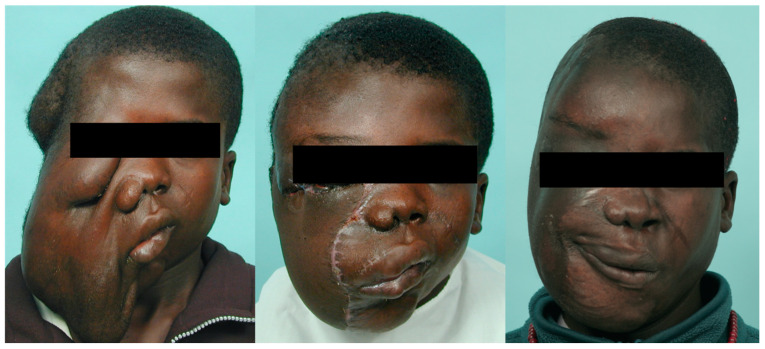
Clinical images of a 15-year-old adolescent boy from Africa who presented with diffuse plexiform craniofacial neurofibromatosis with no ipsilateral facial nerve function and no vision. After preoperative embolization (right superficial temporal, internal maxillary, and facial arteries) and intraoperative ligation of the right external carotid artery, a radical excision of the tumor with orbital repositioning was performed. Microsurgical free flaps (anterior lateral thigh flap and myocutaneous gracilis free flap) were transferred to reconstruct the facial defect. Additional procedures included orbitotomy, skull base tumor removal, and right fronto-orbital craniotomy, with orbital and craniofacial reconstruction and ocular prosthesis placement.

**Figure 5:**
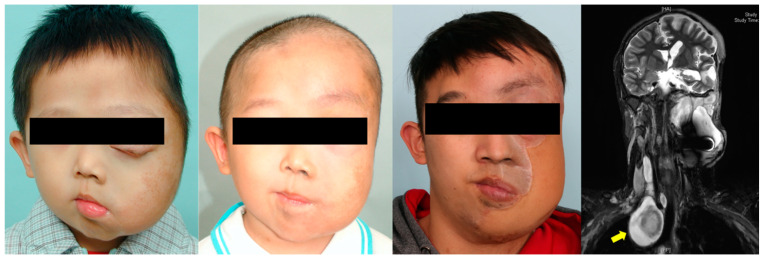
Clinical images of an 11-year-old boy with craniofacial plexiform neurofibromatosis who has been followed up with since the age of 2 and has been receiving orbital and zygomatic repositioning and reconstruction of the orbital roof and temporal bone. Owing to the disease progression, a radical excision was performed after preoperative embolization (branches of the left internal maxillary, lingual, and facial arteries) and total interruption of the left external carotid artery. The soft tissue defect was reconstructed with an anterior lateral thigh flap. Magnetic resonance imaging showed involvement of the left intraorbital, parasellar, parapharyngeal, and posterior pharyngeal regions and adjacent disorganized brain tissues and dural ectasia; craniotomy was performed for skull base and orbital tumor removal. Additional procedures included serial tumor excisions, lip repair, and canthoplasty. Magnetic resonance imaging indicated a mediastinal mass (yellow arrow) with neck swelling, and shortness of breath had developed in recent years. An additional neurofibroma compressing the cervical spine (C2 and C3 level) was also identified.

Seven patients with severe plexiform craniofacial neurofibroma-related functional and aesthetic impairment underwent surgical excisions that required microsurgical free flaps for reconstruction (Figure 3, Figure 4 and Figure 5). Multi-stage and extensive revision procedures were necessarily required [18,19,20,21].

## 9. Discussion

The cohort in our study exemplified the wide spectrum of NF-1 presentation, with various anatomical sites affected by tumors, causing functional and aesthetic impairments in the patients [7,22]. Because NF-1 can be expressed clinically in various manners, differing even between members of the same family carrying the same NF1 mutation, treatment may range from clinical observation and isolated removal of cutaneous tumors to drug-centered therapy for neurological and endocrinological abnormalities and chemotherapy or targeted therapy for conditions such as optic gliomas, malignant peripheral nerve sheath tumors, and GIST [8,9,23,24,25,26,27,28,29]. This long-term study focused on the surgical treatment of patients with NF-1 by using a multidisciplinary team-based approach.

Overall, the number of affected anatomic regions and systems and the number of specialists involved in care were high in our study. We stratified the patients into craniofacial and noncraniofacial groups because it assisted us in surgical decision-making (Figure 1). Moreover, we tested the hypothesis that craniofacial involvement requires an intensive therapeutic approach to restore both functionality and aesthetics of craniofacial structures in the patients. Neurofibroma-related facial disfigurement strongly influences aesthetic appearance and interpersonal relationships, much more so than neurofibromas affecting the trunk or extremity regions [30,31,32,33,34]. Moreover, tumor lesions affecting the cranial nerves or orbital and brain regions can have a major effect on functional activities [5,23,24]. However, we observed no significant differences in the number of surgical interventions and specialists involved in care of patients with and without craniofacial involvement.

Patients with craniofacial plexiform neurofibromas underwent a higher number of surgical interventions and required more specialist treatment during the disease course than did those having craniofacial cutaneous neurofibromas. Plexiform neurofibromas occur less frequently than cutaneous neurofibromas, but plexiform lesions are considered the main source of morbidity in craniofacial NF-1 because these tumors can spread in size, eventually leading to soft tissue hypertrophy, and functional and aesthetic impairments. Therefore, patients with craniofacial plexiform neurofibromas require condition-specific planning for surgical treatment. This particular subset of patients with NF-1 experiences limited improvement after surgical excision of plexiform neurofibromas along with a high risk of perioperative complications and a high recurrence rate, which is frustrating for both the patients and clinicians [25,26].

The most common surgical approach for treating plexiform craniofacial neurofibroma that affects both patients functionality and aesthetics is en bloc translesional excision performed according to the facial aesthetic unit principle and without sacrificing the functional nerves [18]. In our analysis, plastic surgeons, ophthalmologists, neurosurgeons, and radiologists actively participated in the therapeutic management of these patients. Plastic surgeons were responsible for tumor resection and reconstruction. For patients with infiltrating tumors of the orbitotemporal region that cause ptosis, optic nerve compression, or blindness or even involve the brain or skull base, ophthalmologists and neurosurgeons provided the proper management [20,35,36,37]. Proper preoperative diagnosis and planning under the multidisciplinary team-based surgical approach permitted us to successfully maximize the risk-to-benefit ratio for treating these patients with plexiform craniofacial neurofibromatosis and to aim for maximal improvement in the functional and aesthetic outcomes with minimal complications and disruptions in the adjacent functional structures.

Microvascular free tissue transfer following radical resection of large neurofibromas was proven to be a safe and reliable method for the coverage of extensive soft tissue defects [38]. In our experience, despite the successful coverage of large, raw craniofacial wounds, functional reconstruction was not effective. Revision procedures were also required. Alternative forms of soft tissue coverage, including simple skin grafts, could also be considered [18]. In this setting, patients and parents should be advised of the limitations of surgical resection and reconstruction as well as the risk of relapse and malignant transformation [7,8,23]. Moreover, a shared decision-making process between patients, parents, and the members of the multidisciplinary team may assist in defining patients’ expectations and in improving outcomes with treatment and follow-up.

According to a report, 8 to 12% of patients with NF-1 may develop malignant peripheral nerve sheath tumors during their lifetimes [3]. We documented a high incidence rate (16%) of malignant peripheral nerve sheath tumors in our study cohort and detected other malignancies (cerebellar astrocytoma and GIST) during the 25-year period. Because malignant peripheral nerve sheath tumors usually originate from a preexisting plexiform neurofibroma, patients with NF-1, particularly those with plexiform type, should receive regular follow-up to ensure early intervention if necessary.

The limitations of this study include an inherent bias associated with the retrospective design. We provided our current protocol that is the product of 25 years of development, but it should not be considered a unique or static protocol. Other centers engaged in the management of patients with NF-1 should publish their specific protocols and apply, refine, and adjust our protocol to their own environment of care. Future studies should assess further outcome metrics including patient-reported outcomes and total cost effectiveness of adopting a multidisciplinary team-based surgical approach in the management of patients with NF-1. The published protocols and results may provide a basis for enhancing the management of patients with NF-1.

## 10. Conclusions

In this long-term study, we assessed 25 years of surgical experience at a single institution in management of patients with NF-1. Our long-term experience suggests that the multidisciplinary team-based surgical approach should be adopted to provide comprehensive individualized care to patients with NF-1 and warrants its substantial role in treating the patients with complicated plexiform neurofibromas.

## Figures and Tables

**Table 1:** Distribution of patients (*n* = 106) with neurofibromatosis type I according to anatomical region.

Anatomical Region	Patients	Percentage
Skull	18	17.0%
Face	66	62.3%
Orbit	28	26.4%
Brain	11	10.4%
Cranial nerve	1	0.9%
Neck	25	23.6%
Trunk	55	51.9%
Chest wall	2	1.9%
Mediastinum	4	3.8%
Lung	2	1.9%
Extremities	42	39.6%
Spine	19	17.9%
Visceral	1	0.9%

**Table 2:** Specialties involved in surgical care of patients (*n* = 106) with neurofibromatosis type I.

Specialty	Surgical Intervention (Number)	Patients (Number)	Patients (Percentage)
Plastic surgery	160	60	56.6%
Ophthalmology	48	25	23.6%
Neurosurgery	31	22	20.8%
Orthopedics	26	15	14.2%
Dermatology	25	19	17.9%
Radiology	22	13	12.3%
General surgery	13	6	5.7%
Chest surgery	2	2	1.9%
Otorhinolaryngology	2	2	1.9%
Colorectal surgery	1	1	0.9%
Pediatric surgery	1	1	0.9%
